# Placebo Trends across the Border: US versus Canada

**DOI:** 10.1371/journal.pone.0142804

**Published:** 2015-11-25

**Authors:** Cory S. Harris, Natasha K. J. Campbell, Amir Raz

**Affiliations:** 1 Department of Biology, University of Ottawa, Ottawa, Ontario, Canada; 2 Department of Psychiatry, McGill University, Montréal, Québec, Canada; 3 Lady Davis Institute for Medical Research, Jewish General Hospital, Montréal, Québec, Canada; University of Toledo, UNITED STATES

## Abstract

**Background:**

Physicians around the world report to using placebos in a variety of situations and with varying degrees of frequency. Inconsistent methodologies, however, complicate interpretation and prevent direct comparisons across studies. While US- and Canada-based physicians share similar professional standards, Canada harbours a less-litigious universal healthcare model with no formal placebo-related policy—factors that may impact how physicians view and use placebos.

**Methods:**

To compare American and Canadian data, we circulated an online survey to academic physicians practicing in Canada, collected anonymous responses, and extracted those of internists and rheumatologists for comparison to US data obtained through parallel methodologies.

**Results:**

Whereas our data show overall concordance across the border—from definitions to ethical limitations and therapeutic potential—differences between American- and Canadian-based placebo practices merit acknowledgement. For example, compared to 45%-80% among US-based respondents, only 23±7% of Canada-based respondents reported using placebos in clinical practice. However, 79±7% of Canada-respondents—a figure comparable to US data—professed to prescribing at least one form of treatment without proven or expected efficacy. Placebo interventions including unwarranted vitamins and herbal supplements (impure placebos) as well as sugar pills and saline injections (pure placebos) appear more common in Canada, where more doctors described placebos as “placebos” (rather than “medications”) and used them as a “diagnostic” tool (rather than a means of placating patient demands for treatment).

**Interpretation:**

Cross-border variation in the use of clinical placebos appears minor despite substantial differences in health care delivery system, malpractice climate, and placebo-related policy. The prevalence of impure placebos in both Canadian and US clinics raises ethical and practical questions currently unaddressed by policy and warranting investigation.

## Introduction

Whereas prescribing placebos in the clinic generally constitutes “bad professional form”, findings from physician surveys reveal the continued and pervasive use of placebos in clinical practice [1]. The nature and frequency of placebo prescriptions varies across nations perhaps reflecting distinct sociocultural influences. In Germany, for example, formal policy permits circumscribed use of placebos in clinical settings; whereas a small survey among general practitioners in Bavaria reported that 86% of doctors dispensed placebos [2], a larger nation-wide survey among three groups of physicians working in private practice revealed that the use of placebos and non-specific treatments varied as a function of medical specialty and professional attitude [3]. In neighboring Switzerland, conversely, where no placebo-related policies are in place, 57% of doctors administer placebos [4]. Thus, placebo practices can change substantively even across adjacent regions sharing common European sensibilities.

Findings regarding variation in the administration of placebos in North American clinics mostly come from the US and Canada. Surveys conducted in the US estimate that 45–80% of American doctors have prescribed or administered a placebo during routine practice [5, 6]; limited data from Canada suggest similar patterns [7, 8]. However, incompatible methodologies often obfuscate direct comparison between studies and countries [9]. For example, incongruent definitions for terms such as “placebo” and “placebo effect” differentially impact physician responses [1, 10, 11] as do medical specialty, professional outlook, and clinical environment [3, 8, 12]. Compatible methodologies, on the other hand, would permit a more meaningful comparison between physician practices across borders, cultures, health care systems, and public policies.

To elucidate how placebo administration varies across the Canada-US border, we surveyed physicians in Canada about their placebo-related practices and attitudes using a study design affording direct comparison to US data. While Canada- and US-based physicians train and practice medicine following comparable standards, sociocultural factors vary across the border. Canada offers a publicly-funded model of healthcare wherein drugs are often cheaper and litigation is less frequent than in the US [13–15]. Furthermore, Canada lacks a formal policy on the use of placebos in the clinic while the American Medical Association (AMA) upholds circumspect recommendations relative to their German counterparts [16, 17]. Accordingly, we hypothesized that, relative to placebo trends in Canada, physicians in the US would be more conservative toward using placebos in the clinic. Beyond intuition, the rationale for our hypothesis—that placebo prescription rates will be higher in Canada compared to the US—developed against a multifaceted backdrop: from differences in the policies of professional organizations and insurance coverage to clinical outlooks on the use of placebos and non-specific therapies as a coping behavior for difficult and uncertain situations.

## Methods

We designed the investigation based on comparable studies, notably an internet-based survey of academic internists and rheumatologists practicing in the US [5, 12, 18]. Having obtained the necessary ethics approval through the Jewish General Hospital of Montréal Research Ethics Committee (REC), we circulated an online survey to approximately 7600 practicing physicians affiliated with Canadian medical schools. Information Technologies Services at McGill University supported and maintained the online survey, collecting anonymous data confidentially through the Educational Technologies server (Montreal, Canada). As approved by the REC, informed consent was obtained electronically (online) and, serving as a gateway to the survey. To improve compatibility with American data, we extracted the responses of all identifiable internists and rheumatologists. The list of respondents by medical specialty is provided in S1 Table (Supplemental Information). A comprehensive description of our methodology is available elsewhere (see S1 Text).[8]

### Survey

We obtained a copy of the questionnaire employed by Sherman & Hickner, which sampled Chicago-based academic physicians via an online request, and then made minor modifications to adapt it for a Canadian context. The resulting five-minute survey consisted of seven demographic questions followed by 14 placebo-related questions probing topics such as frequency of use and strength of placebo effects in routine care. Our survey is accessible online (http://tinyurl.com/McGillPlacebo - English; http://tinyurl.com/McGillPlaceboQc - French). We hosted the self-report survey using the open source LimeSurvey^®^ web-based application tool and Internet technology to ensure expediency as well as data anonymity. Most questions followed a multiple-choice (closed) format with the option of providing brief text responses (open format) and four questions comprised of a 5-point Likert scale. Participation was voluntary and compensation-free.

### Statistical Analysis

We performed all statistical analyses using SAS^®^ v 9.2 (SAS Institute, Cary, NC), including descriptive statistics, frequency distributions as well as chi-square and Fisher exact tests. American data were extracted from Sherman and Hickers [5].

## Results

Of the 612 anonymous respondents, 18% practice general internal medicine and 14% are subspecialty internists, for a total of 198 qualifying responses for this study, most of which came from physicians practicing in major urban areas (Montreal, Toronto, Ottawa and Vancouver). The average age of respondents is 50 years old and 69% are male. The specific response rate for this subgroup is unknown but likely similar to the study’s global rate (~10%) [8]. All data came from respondents who reported that they were physicians actively seeing patients.

### Defining placebos

When asked which statement(s) best describe(s) their definition of a placebo, 47% of respondents in Canada selected “an intervention that is not expected to have an effect through a known physiologic mechanism”, 43% chose “an intervention not considered to have a ‘specific’ effect on the condition treated, but with a possible ‘unspecific’ effect”, and 33% opted for “an intervention that is inert or innocuous”. Only 2% offered alternative definitions. These results parallel data from the US, where 51%, 37% and 28% of respondents selected each definition, respectively [5].

### Using placebos and placebo-like treatments in the clinic

**Fig 1:**
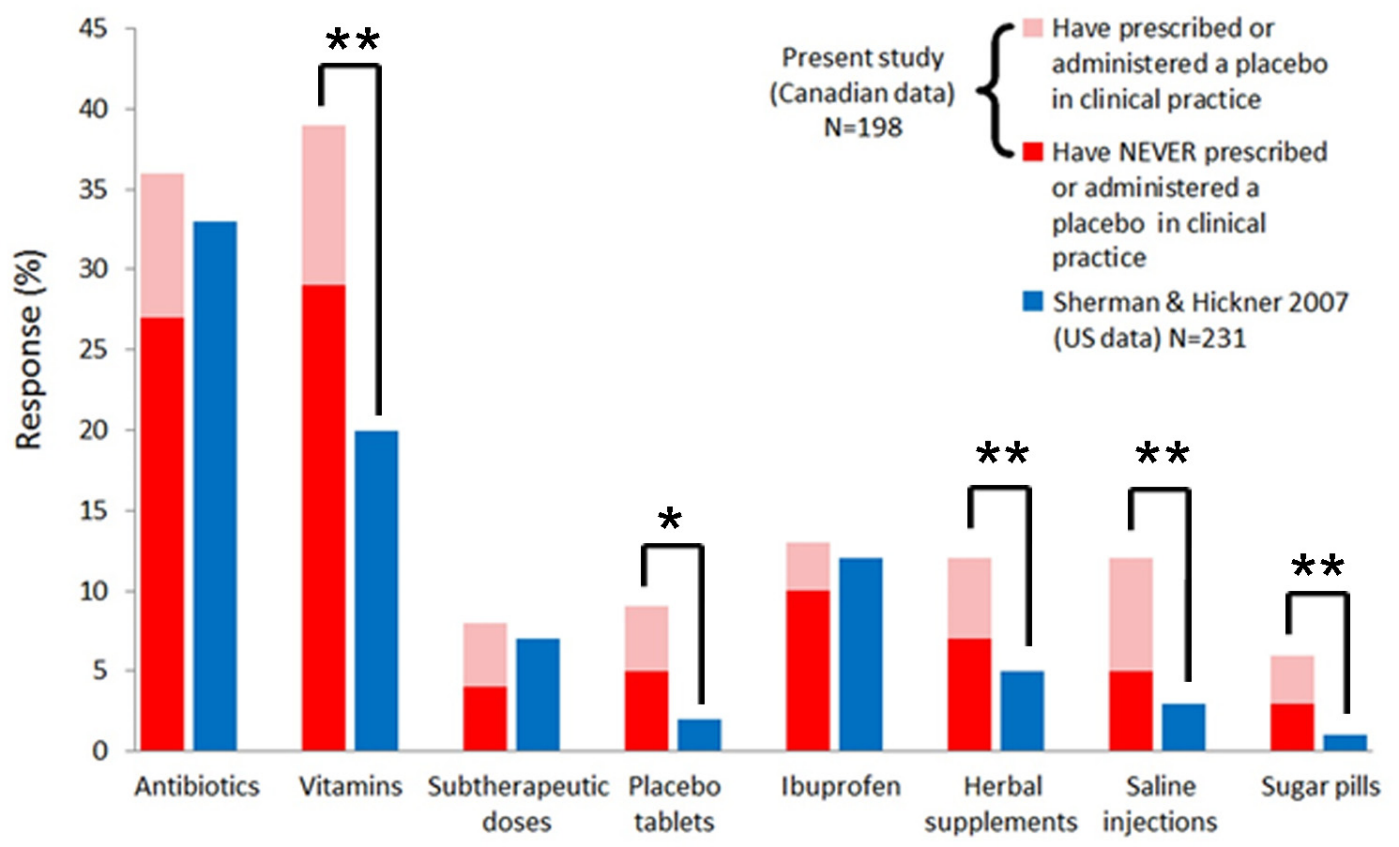
More respondents from Canada reported using various forms of placebo treatments. Response rates among academic internists and rheumatologists from Canada and the US when asked if they have prescribed or given various forms of treatment in situations without demonstrated or expected clinical efficacy. Columns depict the total use of a placebo and placebo-like intervention and colors indicate subgrouping according to responses on survey questions. Shading of Canada-based data reflects the percentage of respondents that, for the following question, reported prescribing/administering (pink) or *never* prescribing/administering (red) a placebo in clinical practice. Stars denote significant differences between groups as determined by chi-square comparisons (★<0.05, ★★<0.005).

The questionnaire did not make an explicit distinction between placebos and placebo-like treatments. Twenty-three percent (±7%) of responding doctors in Canada declared prescribing or administering a placebo in the course of routine clinical practice, at most half the number reported by US studies targeting internists and rheumatologists [5, 6]. A different picture emerges, however, regarding the provision of placebo-like treatments without demonstrated or expected clinical efficacy. While physicians in both countries prescribe unwarranted antibiotics, ibuprofen, and subtherapeutic drug doses at comparable scales, more physicians in Canada prescribe vitamins, herbal supplements, and all forms of pure placebo (e.g., saline injections and sugar pills) (See Fig 1).

Table 1 compares physician responses to questions regarding the circumstances of placebo use, information given to patients, and related ethical limitations. Although differences between nations were scarce, more US-based doctors prescribe placebos to placate “unjustified” demands for medication and fewer utilize placebos as a diagnostic tool. Physicians in the US are also more likely to tell patients they are receiving a medication rather than a placebo, while the opposite is true north of the border. Interestingly, consent for using placebos when openly prescribed to patients—a stipulation explicitly set forth by current AMA policy—pervades more widely in Canada than in the US.

**Table 1:** Placebo attitudes and ways in which physicians would describe placebos to patients.

Question	% of respondents^1^		
	Canada	US^2^	P-value
**I have prescribed or administered a placebo in the following situation(s)**:			
After all clinically indicated treatment possibilities were exhausted	11	11	NS
After "unjustified" demand for medication	8	15	χ2(1) = 5.1 (P = 0.024)
To placate or calm a patient	13	18	NS
To control pain (including in the context of patient-controlled analgesia)	5	6	NS
As a diagnostic tool (e.g., to distinguish between psychogenic and organic causes of symptoms)	10	4	χ2(1) = 6.5 (P = 0.011)
As a supplemental treatment	14	18	NS
For non-specific complaints	12	13	NS
To stop patients from complaining	4	6	NS
**If I were to prescribe a placebo, I would tell the patient that**:			
It is a medication	4	19	χ2(1) = 22.5 (P < 0.0001)
It is a placebo	11	4	χ2(1) = 7.4 (P = 0.007)
It is medicine with no specific effect	6	9	NS
It is a substance that may help and will not harm	35	34	NS
Other	5	33	χ2(1) = 51.6 (P < 0.0001)
**The following statement(s) best describe(s) my position on the use of placebos outside of research**:			
The use of placebos should be categorically prohibited.	9	12	NS
The use of placebos may be permitted if research supports its efficacy.	44	46	NS
The use of placebos may be permitted if the experience of colleagues supports it.	9	9	NS
The use of placebos may be permitted after notifying the patient that he/she is receiving a placebo.	31	21	χ2(1) = 5.7 (P = 0.017)
The use of placebos may be permitted if I anticipate that it will be of benefit to the patient.	37	31	NS

^1^ Sums may exceed 100% due to acceptance of multiple responses.

^2^ reported in Sherman et al., 2008

NS = non-significant, as determined by chi-square test

### Perceived therapeutic potential of placebos

Seventy percent of respondents to our survey agreed that the placebo effect is real, which differs significantly from the 95% agreement among US-based doctors. However, beliefs about the role of psychological and biological (or “biochemical” in the US study) factors in placebo effects were nearly identical across countries, as were opinions regarding the benefits proffered by meditation and yoga, social support systems, and prayer or spirituality. Compared to US data, physicians in Canada ascribe greater therapeutic potential to expectation and belief, good emotional health, and doctor-patient rapport, but less potential to biofeedback (Table 2). When probed about possible benefits of placebo treatments for different health problems, Canada- and US-based doctors shared similar views on the relative potential of placebo for conditions such as pain versus cancer, but doctors practicing in the US are notably more skeptical; fewer attribute both psychological and physiological benefits to placebos and more believe placebos offer no benefit for all listed categories (Table 2).

**Table 2:** Comparison of Canadian and American response distributions for questions regarding the therapeutic mechanisms and potential of placebos and placebo-like therapies.

**A. What benefits do you think placebo treatments can have for the following health problems?**		
	**Both PSYC and PHYS**	**Neither PSYC nor PHYS**
**Condition**	**Canada (%) / US (%)**	**Canada (%) / US (%)**
Pain	59 / 40*	9 /15
Mental or emotional disorders	49 / 37*	6 / 14*
Sexual dysfunction	48 / 33*	8 / 18*
Recovery from drug addiction	47 / 32*	13 / 24*
Gastrointestinal disorders	44 / 35*	11 / 22*
Neurological disorders	38 / 28*	17 / 33*
Immune problems or allergies	31 / 28	29 / 45*
Cardiovascular disorders	30 / 23*	28 / 47*
Cancer	29 / 23	19 / 33*
Viral infections	20 / 20	30 / 38
**B. No consensus has been reached regarding the following categories**.		
** What types of benefits do you think they can have?**		
	**Both PSYC and PHYS**	**Neither PSYC nor PHYS**
**Condition**	**Canada (%) / US (%)**	**Canada (%) / US (%)**
Meditation, yoga or relaxation techniques	86 / 83	1 / 1
Good emotional health	82 / 74*	1 / 0
Social support system	69 / 65	1 / 0
Biofeedback	65 / 76*	7 / 4
Doctor-patient rapport	64 / 51*	2 / 6
Complementary and alternative medicine	64 / 69	6 / 6
Expectation or belief	61 / 48*	3 / 3
Prayer or spirituality	58 / 53	4 / 3
Interior design of health care environment	34 / 37	14 / 15

* indicates significant differences between Canada and US data as determined by chi-square comparisons (p < 0.05)

PSYC = psychological benefit; PHYS = physiological benefit

## Interpretation

Comparison of data concerning the clinical use of placebos in the US vs. Canada reveals remarkable concordance between the two sides of the border. The most striking discrepancy underlines that only 23% respondents in Canada attest to using a placebo in the clinic—far fewer than in the US and among the lowest rates reported in nations surveyed over recent years [1]. In contrast, the prevalence of placebo-like treatments in Canadian clinics matches, even exceeds, that of the US: 79% of Canada-based doctors prescribe at least one form of treatment without expected or proven efficacy. Whether due to vague definitions of what constitutes a placebo, reluctance to associate one’s practice with placebo, or other reasons, physicians in Canada hardly view themselves as placebo prescribers. For example, nearly 40% admitted to prescribing “unwarranted” vitamins yet ~75% subsequently reported never having prescribed a placebo (Fig 1).

Paradoxically, more American doctors believe the placebo effect is real yet more Canadians believe placebo treatments, expectations, and doctor-patient rapport can benefit patients both psychologically and physiologically (Table 2). Relative medical litigiousness and treatment costs may underlie why US-based doctors are more likely to describe placebos as medication and prescribe them to placate patients while doctors in Canada more commonly prescribe (pure) placebos transparently. Such nuances, however, clearly require more (qualitative) research to untangle and interpret.

Beyond any difference, the degree of concordance between American and Canadian data is striking—from definitions to clinical practices to perceived therapeutic potential. In addition to prescribing placebo treatments, the majority of physicians in North America believe that the practice is ethically permissible in certain situations and can elicit real therapeutic benefits for patients. Consistent with international data, North American doctors turn to pure placebos far less frequently than to impure placebos. This trend is problematic: while impure placebos continue to raise ethical concerns, physicians neither perceive such interventions as placebo treatment nor do they have guidance, including from the AMA, concerning the use of impure placebos in clinical situations [9, 17].

### Caveats and Limitations

We would like to acknowledge a few overarching weaknesses of the present study. First, our response rate, although acceptable for online surveys [19, 20], is likely lower than that used by Sherman & Hickner in their US survey. Second, despite largely compatible methodologies, low response rates and the different geographical distribution of respondents limit the interpretation of direct comparison between studies. Response representativeness denotes more than response rate [21, 22] and the present demographic data are congruent with data drawing on more than 62,000 physicians practicing in Canada [23]. In this regard, our findings likely represent a valuable contribution to preliminary investigations of placebo use among physicians and their beliefs about placebo mechanisms and effectiveness. However, the fact that our demographic data are similar to national numbers hardly implies that the use and beliefs of placebos among respondents would be representative for physicians practicing in Canada. For example, we have already shown that placebo use and beliefs among Canadian psychiatrists differ from those of other physicians [8]. Moreover, other researchers have reported that placebo use and beliefs differ among different types of physicians and settings [3, 12]. Third, our questionnaire allowed multiple appellations for placebos, which may drive respondents to construe and interpret questions in different ways.

## Conclusion

Despite fundamental differences between US and Canadian health care, North American doctors share common views and clinical practices when it comes to placebo. In line with physician attitudes in Europe and Israel, few North American physicians appear to favor the elimination of placebos from clinical practice and most acquiesce to the clinical merits associated with placebos. Still, ethical concerns as well as limited clinical guidelines and relevant medical training continue to obviate a medical grey zone capable of enhancing (or diminishing) the quality of health care. Doctors use placebos and will continue to do so, whether in the US, Canada, or elsewhere. To ensure the safe, effective, and ethical use of placebos we must educate practitioners in placebo science and extend our debates and policies beyond sugar pills and saline injections. Unlike the restrictive position of the AMA in the US or the liberal stance of its sister organization in Germany, Canada currently maintains no clear policy on the use of placebos in clinical care. While some propose that this conundrum ought to lead open discussion, instate clear guidelines, and come up with specific protocols to govern the use of placebos, others suggest that the lack of a precise policy affords clinical latitudes that may benefit patients [24]. Meanwhile, placebos remain the kind of clinical intercession we can really believe in.

## Supporting Information

S1 Table List of respondents by medical specialty. The frequency, percent, cumulative frequency, and cumulative percent of participants that indicated their primary medical specialty, listed in alphabetical order of specialty. Respondents reported specialties highlighted in yellow (General and Internal Medicine) and in red text (Subspecialty of Internal Medicine) were included in the presented analysis. Other medical specialists were excluded.
(DOCX)

S1 Text Raz et al. Can J Psychiatry, 2011. The data presented in the submitted manuscript were extracted from a larger dataset that was used to publish a comparison of placebo practices and perceptions between psychiatrists and other physicians in Canada. The extracted data were re-analyzed relative to U.S. data. No results, data, or figures were duplicated from this related publication.
(PDF)
