# Supplementary material for: Placebo Trends across the Border: US versus Canada
Source: PLoS One. 2015 Nov 25;10(11):e0142804. doi: 10.1371/journal.pone.0142804 (PMC4659632; doi:10.1371/journal.pone.0142804)
Supplement: S1 Table — The frequency, percent, cumulative frequency, and cumulative percent of participants that indicated their primary medical specialty, listed in alphabetical order of specialty. Respondents reported specialties highlighted in yellow (General and Internal Medicine) and in red text (Subspecialty of Internal Medicine) were included in the presented analysis. Other medical specialists were excluded. (DOCX) [file pone.0142804.s001.docx]

| **TSpecialty** | **Frequency** | **Percent** | **Cumulative Frequency** | **Cumulative Percent** |
| --- | --- | --- | --- | --- |
| **Anesthesiology** | 29 | 4.74 | 29 | 4.74 |
| **Cardiology** | 7 | 1.14 | 36 | 5.88 |
| **Clinical Pharmacology** | 1 | 0.16 | 37 | 6.05 |
| **Dermatology** | 4 | 0.65 | 41 | 6.70 |
| **Emergency** | 8 | 1.31 | 49 | 8.01 |
| **Endocrinology** | 8 | 1.31 | 57 | 9.31 |
| **Ethics** | 1 | 0.16 | 58 | 9.48 |
| **Family Medicine** | 39 | 6.37 | 97 | 15.85 |
| **Gastroenterology** | **6** | 0.98 | 103 | 16.83 |
| **General Medicine** | 3 | 0.49 | 106 | 17.32 |
| **Geriatrics** | 5 | 0.82 | 111 | 18.14 |
| **Hematology** | **4** | 0.65 | 115 | 18.79 |
| **Hematology/Oncology** | **1** | 0.16 | 116 | 18.95 |
| **Immunology** | **3** | 0.49 | 119 | 19.44 |
| **Infectious Diseases** | **5** | 0.82 | 124 | 20.26 |
| **Internal Medicine** | 53 | 8.66 | 177 | 28.92 |
| **Medical Biochemistry** | 2 | 0.33 | 179 | 29.25 |
| **Medical Genetics** | 1 | 0.16 | 180 | 29.41 |
| **Medical Imaging** | 1 | 0.16 | 181 | 29.58 |
| **Medicine** | 1 | 0.16 | 182 | 29.74 |
| **Nephrology** | **5** | 0.82 | 187 | 30.56 |
| **Neurology** | **22** | 3.59 | 209 | 34.15 |
| **None** | 1 | 0.16 | 210 | 34.31 |
| **OBGYN** | 16 | 2.61 | 226 | 36.93 |
| **Obstetrics** | 1 | 0.16 | 227 | 37.09 |
| **Oncology** | **1** | 0.16 | 228 | 37.25 |
| **Ophthalmology** | 6 | 0.98 | 234 | 38.24 |
| **Orthopedics** | 4 | 0.65 | 238 | 38.89 |
| **Otolaryngology** | **5** | 0.82 | 243 | 39.71 |
| **Pain Management** | 1 | 0.16 | 244 | 39.87 |
| **Pediatrics** | 66 | 10.78 | 310 | 50.65 |
| **Pharmacology** | 1 | 0.16 | 311 | 50.82 |
| **Physical Medicine and Rehabilitation** | **1** | 0.16 | 312 | 50.98 |
| **Psychiatry** | 257 | 41.99 | 569 | 92.97 |
| **Pulmonary** | **1** | 0.16 | 570 | 93.14 |
| **Pulmonary Medicine** | **2** | 0.33 | 572 | 93.46 |
| **Radiation Oncology** | 6 | 0.98 | 578 | 94.44 |
| **Radiology** | **1** | 0.16 | 579 | 94.61 |
| **Rehabilitation** | **1** | 0.16 | 580 | 94.77 |
| **Respirology** | **4** | 0.65 | 584 | 95.42 |
| **Rheumatology** | **5** | 0.82 | 589 | 96.24 |
| **Surgery** | 18 | 2.94 | 607 | 99.18 |
| **Urogenital Medicine** | **1** | 0.16 | 608 | 99.35 |
| **Urology** | **4** | 0.65 | 612 | 100.00 |
