# Supplementary material for: Placebo Trends across the Border: US versus Canada
Source: PLoS One. 2015 Nov 25;10(11):e0142804. doi: 10.1371/journal.pone.0142804 (PMC4659632; doi:10.1371/journal.pone.0142804)
Supplement: S1 Text — The data presented in the submitted manuscript were extracted from a larger dataset that was used to publish a comparison of placebo practices and perceptions between psychiatrists and other physicians in Canada. The extracted data were re-analyzed relative to U.S. data. No results, data, or figures were duplicated from this related publication. (PDF) [file pone.0142804.s002.pdf]

Placebos in Clinical Practice:

Comparing Attitudes, Beliefs, and Patterns of Use between

Academic Psychiatrists and Non-Psychiatrists

Amir Raz, Ph.D.[1]; Natasha Campbell, B.Sc.[2]; Daniella Guindi, B.Sc.[3];

Christina Holcroft, Sc.D.[4]; Catherine Déry, D.E.C.[5]; Olivia Cukier, B.A.[6]

[1]Corresponding Author. McGill University, Departments of Psychiatry, Neurology and Neuroscience, and Psychology, McGill University and the Jewish General Hospital, Montreal Neurological Institute, 3775 University Street, Montreal, Québec, Canada. E-mail: amir.raz@mcgill.ca

[2]MSc Student, Department of Psychiatry, Faculty of Science, McGill University, Montreal, Québec, Canada

[3]MD Student, Faculty of Medicine, McGill University, Montreal, Québec, Canada

[4]Statistical Consultant, Jewish General Hospital, Centre for Clinical Epidemiology and Community Studies, Montreal, Québec, Canada

[5]Nursing Student, Université de Sherbrooke, Sherbrooke, Québec, Canada

[6]DMD Student, Faculty of Dentistry, McGill University, Montreal, Québec, Canada

DRAFT

Abstract

Controversial and ethically tenuous, the use of placebos is central to medicine but even more pivotal to psychosocial therapies. Scholars, researchers, and practitioners largely disagree about the conceptualization of placebos. While different professionals often confound the meanings of placebo effects with placebo responses, physicians continue to prescribe placebos as part of clinical practice. The present study aims to review attitudes and beliefs concerning placebos outside of clinical research. Herein we compare patterns of placebo use reported by academic psychiatrists with those reported by physicians from different specialties across Canadian medical schools. Using a web-based tool, we circulated an online survey to all 17 Canadian medical schools with a special emphasis on psychiatry departments therein and in university-affiliated teaching hospitals. A variation on earlier efforts, our 5-minute, 21-question survey was anonymous.

Six-hundred-and-six respondents, 257 of whom were psychiatrists, completed our online survey. Our analysis revealed that psychiatrists prescribed significantly more subtherapeutic doses of medication than physicians in other specialties, although about 20% of both psychiatrists and non-psychiatrists prescribed placebos regularly as part of routine clinical practice. Compared to 6% of non-psychiatrists, however, only 2% of psychiatrists deemed placebos of no clinical benefit. In addition, more than 60% of psychiatrists either agreed or strongly agreed that placebos had therapeutic effects relative to fewer than 45% of other practitioners.

Findings from this Pan-Canadian survey suggest that, compared to other physicians, psychiatrists seem to better value the influence placebos wield on the mind and body and maintain more favourable beliefs and attitudes towards placebo phenomena.

DRAFT

Keywords: placebos; clinical practice; Canadian physicians; academic psychiatry; online surveys.

Highlights:

1. The majority of physicians acquiesce to the effects of placebos, yet they seem equivocal regarding a common placebo description.
2. Probably because they construe them as therapeutic, psychiatrists seem to administer significantly more subtherapeutic doses of medication compared to non-psychiatrists.
3. Our findings likely represent a valuable contribution to preliminary investigations of placebo use among physicians and their beliefs about placebo mechanisms and effectiveness.

DRAFT

Congruent with the working definition assumed in the high-powered world of pharmacology, most physicians construe placebos as the non-specific effects of medical treatment that, in clinical trials, must be controlled for to assess the specific effects of new (drug) interventions.[1] Placebo-like treatments, accordingly, refer to any short-term or illusory impression of improved health that some patients experience when they receive what appears to be effective treatment but actually isn't for the condition being treated. As such, the placebo effect is a powerful mind–body phenomenon with a specific underlying biology that health professionals should investigate and exploit.[2]

Exemplifying the link between psychosocial factors and physiological processes, placebos are central to medicine[3] but even more pivotal to psychiatry.[4] Furthermore, placebos bind behavioural science to the techniques of neuroscience.[5-8] Several scholars grant placebos a prominent place in clinical psychiatry[4, 9, 10] and mounting evidence suggests a large placebo component even in drugs forming the backbone of biological psychiatry.[11-17] Together with the majority of physicians, however, most modern psychiatrists find the science of placebos difficult to swallow.[18]

Shrouded in a checkered history, placebo use in a therapeutic context remains controversial. Indeed, in 2006 the American Medical Association (AMA) cautioned that "[p]hysicians may use [a] placebo for diagnosis or treatment only if the patient is informed of and agrees to its use".[19] The AMA admonition followed a controversial meta-analysis of clinical trials suggesting that placebo effects are either minimal or non-existent and that "outside the setting of clinical trials, there is no justification for the use of placebos".[20] Multiple researchers have critiqued many aspects of this controversial meta-analysis,[21-24] and reanalysis of the data yielded findings of a "robust" placebo effect[25] resulting in a flurry of rebuttals and debates.[26-28] The charged AMA

DRAFT

statement, however, still colours the views of many clinicians.[29] Despite subsequent discussions of this issue in bioethical circles,[30, 31] the AMA tenor still guides many of the assumptions that the medical community maintains about placebos.[18] The Canadian Medical Association is yet to draft a formal policy regarding the use of placebos in clinical practice.

The placebo flame has been recently rekindled with reports of placebos being dispensed as part of routine care.[32] Publications concerning placebos now span research studies,[12] reviews,[33, 34] books,[8, 35] and popular media coverage,[36, 37] including legal scholarship[29] and social science.[38-41] The widespread use of placebos in clinical practice has been demonstrated in a recent survey of internists and rheumatologists in the United States (US)[42] revealing that of the 679 physicians who replied, more than half said they prescribed "placebo treatments" every now and then, and that they deemed the practice ethical. About 40% of respondents reported they used painkillers or vitamins as placebos and 13% acknowledged using antibiotics and sedatives for this purpose; barely 3% said that they used sugar pills. Over two-thirds, however, reported that rather than calling them placebos they described the pills to patients as "a potentially beneficial medicine or treatment not typically used for their condition." Five percent of physicians reported telling their patients that they were receiving a placebo and 62% believed that prescribing placebos was an ethically acceptable practice.

A number of similar studies have been conducted in select geographic locations outside of Canada.[43-49] For example, a Danish study reported that 86% of general practitioners have used placebos at least once, with 48% using placebos more than 10 times in the previous year.[47] A separate study from Israel found that 60% of respondents prescribed placebos.[48] Among those, 62% reported that they prescribed placebos as often as once a month. Another US study targeting academic physicians in the Chicago area reported that placebos were being used in

everyday clinical practice.[49] Forty five percent of physicians reported that they had used placebos and 96% of physicians believed that placebos had a therapeutic effect. The sparse data from physicians practicing in Canada motivated us to probe the role of placebos in clinical care. Here we show results from an online survey comparing academic psychiatrists to other academic physicians across Canada. Because placebo responses and effects often occur more readily when the endpoint of treatment is a change in behaviour,[50] we expected psychiatrists to differ from other physicians. Compared to non-psychiatrists, therefore, we hypothesized that psychiatrists would display better placebo knowledge, different beliefs, more tolerant attitudes, and heightened patterns of use. In addition, we expected sex-based differences between male and female physicians. We envisaged that female psychiatrists would have a tendency to me more compassionate toward and more innovative about treating their patients than would male psychiatrists. Accordingly, we hypothesized that male psychiatrists, relative to female psychiatrists, would be less likely to integrate placebos into their medical practice.

Method

Using the open source LimeSurvey® web-based application tool, we designed our survey to collect self-report information concerning placebos in clinical practice. Our 5-minute survey implemented a number of computerized checks to preclude invalid data, and ensured expediency as well as data anonymity. Following seven demographic questions, fourteen placebo questions covered topics such as strength of placebo effects and their use outside clinical trials. Most questions followed a multiple-choice (closed) format with the option of providing brief text responses (open format). A few questions featured a 5-point Likert scale. Participation was voluntary and we offered no monetary compensation to respondents. An adaptation fromearlierquestionnaires,[47-49] the current survey remains available online at the following links:

English – http://tinyurl.com/McGillPlacebo

French – http://tinyurl.com/McGillPlaceboQc

Procedure

We circulated our survey to academic physicians by contacting all medical schools across Canada. We broached our bilingual research project with each of the 17 deans of medicine and asked that they consider distributing our survey to academic physicians under their administrative auspices. With three deans abstaining (Université de Montréal, Université Laval, Université de Sherbrooke) for unspecified reasons, we estimate that our survey reached approximately 7600 academic physicians from the remaining 14 schools. In a separate effort to target psychiatrists, we similarly contacted the chairs of all psychiatry departments across Canada and everyone responded favourably.

Medical schools and psychiatry departments that did not reply to our initial e-mail received follow-up phone calls. E-mails to the deans and chairs provided English-French information regarding the nature and relevance of the study, as well as the institutional ethics approval. We requested that the deans encourage all physicians to complete the web-based survey. For the chairs of psychiatry, the e-mail also outlined the importance of placebos in psychiatry and the value of receiving feedback from practicing academic psychiatrists.

A brief e-mail, crafted for the physicians, described the research study and provided live links to the survey in both French and English. We informed participants that the survey was completely anonymous. In accordance with certain provincial constraints (e.g., section 30.1 of the BC Freedom of Information and Protection of Privacy Act) we stored and accessed all survey information in Canada. Based in Montreal, McGill University's Information Technologies

DRAFT

Services provided support and maintenance of the online survey and ascertained data confidentiality through the Educational Technologies team.

Statistical Analysis

We analyzed the data using descriptive statistics and frequency distributions using SAS statistical software, version 9.2 (Statistical Analysis Systems, SAS Institute Inc, Cary, NC), including Chi-square and Fisher exact tests.

Results

General

Respondents comprised 606 academic physicians, 257 (42.41%) of whom were psychiatrists. Male and female respondents represented 65% and 35% of the sample, respectively. Age ranged from 24 to 88 years (median=52; mean=51.1).

Definitional Discrepancies

Table 1 shows statistically significant differences between psychiatrists and other physicians concerning characterizations of placebo.

*** INSERT TABLE 1 ABOUT HERE ***

Administration in Clinical Practice

About 20% of physicians – be they psychiatrists or non-psychiatrists – reported that they had either prescribed or administered a placebo in the course of routine clinical practice. Only 2% of psychiatrists reported that placebos had no clinical benefit compared to 6% of other physicians ($\chi^2(1) = 4.72$, $P = 0.03$). Forty-three percent of psychiatrists indicated that the use of placebos might be permitted after notifying patients that they are receiving a placebo whereas 28% of non-psychiatrists concurred ($\chi^2(1) = 14.36$, $P < 0.001$).

DRAFT

Figure 1 compares how psychiatrists and other physicians indicated their use of “unwarranted” treatments. For example, 38% of psychiatrists reported giving subtherapeutic doses of medication to their patients compared to 6% of non-psychiatrists ($\chi^2(1) = 97.36$, $P < 0.001$). In addition, 16% of psychiatrists, relative to 9% of the remaining sample, have prescribed prefabricated placebo tablets ($\chi^2(1) = 6.39$, $P = 0.01$). Table 2 outlines what physicians contemplating a hypothetical situation involving placebo administration would say to their patients.

*** INSERT FIGURE 1 & TABLE 2 ABOUT HERE ***

Strength of Placebo Effects

Psychiatrists, compared to non-psychiatrists, were more likely to rate placebos as having powerful therapeutic effects on children (31% to 16%, respectively), undereducated patients (25% to 15%), suggestible patients (70% to 60%) and patients from non-Western cultural backgrounds (9% to 3%). Unlike group differences for children ($\chi^2(1) = 17.65$, $P < 0.001$), suggestible patients ($\chi^2(1) = 5.78$, $P = 0.02$) and non-Western patients ($\chi^2(1) = 12.36$, $P < 0.001$), differences in ratings for undereducated patients ($\chi^2(1) = 9.64$, $P = 0.002$) were due to response variation between 27% of male psychiatrists and 13% of other male practitioners ($\chi^2(1) = 11.95$, $P < 0.001$).

Approximately 18% of female physicians rated placebos as having powerful therapeutic effects on women; however, discrepancies in agreement existed between 26% of male psychiatrists and 17% of male non-psychiatrists ($\chi^2(1) = 4.40$, $P = 0.04$).

Different levels of agreement arose between psychiatrists and non-psychiatrists when responding to the statement “the placebo effect is real” and “placebos have therapeutic effects”. Specifically, among psychiatrists over 77% agreed or strongly agreed that “the placebo effect is

DRAFT

real" as compared to less than 68% of other physicians ($\chi^2(1) = 6.86$, $P = 0.009$). Figure 2 displays the response distribution to the assertion "placebos have therapeutic effects."

*** INSERT FIGURE 2 ABOUT HERE ***

Disparities among psychiatrists and other physicians occurred when considering the effect of medication colour ($\chi^2(4) = 20.60$, $P<0.001$), personality and bedside manner of the physician ($\chi^2(4) = 10.34$, $P = 0.04$), and the clinician's belief in treatment effectiveness ($\chi^2(4) = 19.60$, $P<0.001$). Distributions weighed more towards psychiatrists believing that these factors influenced a patient's response to medication. However, some of these differences between psychiatrists and non-psychiatrists existed due to differences between male and female practitioners; for example, medication colour and physician beside manner ($\chi^2(4) = 17.93$, $P = 0.001$, and $\chi^2(4) = 13.16$, $P = 0.01$, respectively).

Approximately 90% of physicians reported that psychological factors played a role in explaining how patients may benefit from a placebo. In contrast, more psychiatrists (47%) compared to the rest of the sample (25%) accounted for biological factors ($\chi^2(1) = 33.14$, $p< 0.001$). About 70% of females reported that the mind-body connection was at play; however, a difference exists between the 67% of male psychiatrists and 56% of male non-psychiatrists that agreed ($\chi^2(1) = 4.77$, $P = 0.03$).

Health Benefits of Placebos and Other Alternative Methods

A difference emerged between psychiatrists and other physicians when asked about the benefits placebos may have in a variety of health problems, as displayed in Table 3, Item # 20. In addition, Item # 21 shows the types of benefits various alternative methods may have, according to psychiatrists and non-psychiatrists.

*** INSERT TABLE 3 ABOUT HERE***

DRAFT

Discussion

Compared to other medical specialties, psychiatrists appear more complaisant in their attitudes and beliefs towards placebos. Although about 20% of psychiatrists – comparable to other physicians – responded affirmatively to the question "Have you ever prescribed a placebo in the course of routine clinical practice?" psychiatrists reported using subtherapeutic doses of medication significantly more than non-psychiatrists (see Figure 1). This spike in the administration of subtherapeutic drugs was prevalent irrespective of sex and age of psychiatrist. Fewer psychiatrists (2%), compared to non-psychiatrists (6%), reported that placebos had no clinical benefit. This finding suggests that psychiatrists may better appreciate the clinical merits of using placebos in routine care. Our findings, moreover, suggest that physicians may only partially heed the AMA admonition.[20] About 90% of respondents agreed that psychological factors play a role in explaining placebo benefits. More psychiatrists (47%) than non-psychiatrists (25%), however, reported that biological factors explain how placebos may benefit patients. This attitude extends to other categories (see Table 3). For example, over 95% of psychiatrists report believing that relaxation techniques have both psychological and physiological benefits. Furthermore, findings indicate that psychiatrists appreciate the effects placebos can engender in a range of disorders (see Table 2). Because of their continuous exposure to the effects of a disrupted mind on health, psychiatrists may better appreciate the therapeutic effects of placebos.

Figure 1 shows that two variations on the placebo theme seem palatable as treatment options in situations without expected clinical efficacy. One variation refers to pseudoplacebos – placebo-like interventions that may be active in principle but unlikely effective for the condition being treated – which comprise such treatments as vitamins for chronic insomnia.[51] In the present study

DRAFT

we show that the use of pseudoplacebos is rampant in clinical practice. This trend is increasingly prevalent probably because using pseudoplacebos reduces some of the logistical and ethical problems associated with inert placebo administration. In other words, ethical concerns appear less tenuous when a physician prescribes an active substance, albeit speciously.[52, 53] Figure 1outlines how psychiatrists as well as non-psychiatrists prescribe a variety of pseudoplacebos, including vitamins, herbal supplements, and other treatments. This figure also demonstrates that non-psychiatrists prescribe significantly more antibiotics, ibuprofen, and saline infusions than psychiatrists. In line with the disorders that they see and treat, psychiatrists should seldom prescribe patients with antibiotics and ibuprofen; however, they do appear to prescribe more prepared placebo pills (e.g., commercially available lactose pills) relative to the other responding physicians.

A second variation has to do with the notion of a superplacebo– a treatment that is an actual placebo wherein neither the prescribing practitioner nor the receiving patient is aware of the absence of evidence to recommend it therapeutically.[54] Having gleaned the insights of multiple clinical psychiatrists, our findings suggest that at least some psychiatrists view prescribing subtherapeutic doses of psychiatric medication as clinically therapeutic.[55] As a case in point, in the 1980s, haloperidol dosing of up to 100 mg/day was not unusual and a dose of 2-4 mg/day would have been considered “homeopathic” if not a downright placebo. Subsequent studies, however, have suggested even such low doses as potentially therapeutic. Thus, when administering subtherapeutic doses of medication, at least some psychiatrists may be under the impression that they are instigating an effect that may have therapeutic value.[51]

Placebo confusion appears deeply-entrenched because although nearly half of physicians reported that they “would never give a placebo outside of a clinical research trial” (on Item #11),

DRAFT

many more indicated that they have prescribed placebo-like treatments (on Item #9). It is likely that fewer physicians explicitly report to prescribing placebos in clinical practice because such admission implies bad professional form: congruent with the AMA policy, the dominant view among medical researchers and clinicians deems placebo administration ethically problematic and most doctors feel effectively prohibited from using placebos in clinical practice.

Clinicians who purposefully prescribe unwarranted treatments run the risk of both legal and ethical transgressions. Prescribing treatments without demonstrated clinical efficacy is tenuous; however, at least some psychiatrists appear to believe that subtherapeutic doses have therapeutic effects. Two common scenarios leading to the prescription of subtherapeutic doses include: 1. the practice of "start-low-and-go-slow" – psychiatrists often start patients on an ineffective dose of medication that they intend to gradually increase, but some patients display improvement at doses that remain far below a standard pharmacological threshold (e.g., prescribing 25mg of chlorpromazine while the recommended dose is 600-1000mg).[56] 2. Receiving new patients that are already taking subtherapeutic doses of medication, the receiving psychiatrist continues to prescribe the same low dose because the patient appears to benefit.

Either deliberately or unwittingly, psychiatrists appear to be savvy placebo users. A recent meta-analysis, for example, reported that antidepressants – flagship drugs of modern psychiatry – were largely comparable to placebos for most individuals suffering from depression; antidepressants were clinically superior to placebos in people with extreme depression only.[57] Although this controversial account has been the focus of heated debates, additional data have supported the notion that antidepressants are certainly less effective than we have been led to believe, and in many cases possibly as effective as inert placebos.[11, 58, 59] Other examples span polypharmacy – using more than one drug for the same underlying condition without evidence-based research to

support it – and off-label medications – using a drug for a purpose different from its intended indication or using an atypical dosage-related interpretation. These increasingly rampant methods of treatment, especially in the elderly,[60] appear to gel with recent studies reporting a dramatic surge in placebo response since the 1980s.[61]

That psychiatrists prescribe more subtherapeutic doses than other physicians is contrary to accounts suggesting that general practitioners are more likely to prescribe such doses;[62-64] however, it supports the notion that psychiatrists prescribe a broad range of doses. The term subtherapeutic has many interpretations and in our survey we left those to the discretion of the physician. Whereas some physicians may interpret subtherapeutic through the lens of drug blood-levels and as such, a function of the patient's metabolism, others construe subtherapeutic dose as any prescription that is below the recommended therapeutic level. Most physicians surely appreciate that homeopathy is incongruent with some basic principles of modern science and likely distinguish subtherapeutic dosage from homeopathic quantities. With over 35% of responding psychiatrists prescribing what they believe to be subtherapeutic doses, however, further investigation should elucidate this lacuna.This issue becomes all the more complicated when even "therapeutic" doses of antidepressants seem to resonate, at least in large part, with the appellation of placebos.[11, 12, 58]

Limitations and Caveats

In addressing the relative merits and drawbacks of Internet surveys we refer the reader to a recent special issue in *Public Opinion Quarterly* (Vol. 72, No. 5, 2008). On the one hand, a few of these shortcomings include the challenge of drawing representative samples of the general population; dealing with the issue of people without Internet access; and minimizing the potential for non-response bias. These potential caveats weaken the generalizability of Internet

DRAFT

surveys, especially those focused on broad and diffuse populations. On the other hand, Internet surveys entail advantages, such as reduced social desirability, turning them into valuable research tools under certain conditions.[65] In this section, we provide a detailed account showing that these latter conditions apply to the present study.

Unlike typical surveys of specifically named persons requiring a response rate of at least 60%, the present Internet survey targeted academic physicians without referring to specific individuals. As such, adhering to the definitions and metrics proposed by the American Association for Public Opinion Research is unsuitable.[66-68] In addition, response rates of online surveys using email invitations outperform other electronic media, such as mobile short messaging service, without compromising the sample composition of respondents.[69] On the other hand, response rates for Internet surveys such as the present study differ from mail surveys,[70-73] with characteristic values falling below 10%.[74-77] Our calculations show that the response rate in the present study was about 10%. Response representativeness, however, denotes more than response rate.[78, 79] The present demographic data are congruent with data drawing on more than 62,000 physicians practicing in Canada.[80] Our findings, therefore, likely represent a valuable contribution to preliminary investigations of placebo use among physicians and their beliefs about placebo mechanisms and effectiveness.

The advantages of web-based surveys are multiple: they are expedient, allowing for efficient data collection and timely results; they permit casting a wide net while reducing the cost relative to the sample size;[71] they eliminate the need for a full mailing address, and thus provide respondents with a guarantee of anonymity.[81] Consequently, respondents benefit from social advantages such as an increased willingness to answer charged (e.g., socially threatening)

DRAFT

questions[82] as well as a reduction, or elimination, of social desirability effects.[83] This feature is of special importance when addressing the ethically tenuous topic of placebos in clinical care. The disadvantages of web-based surveys include several aspects. For example, they exclude responses from individuals without internet access thereby introducing coverage error.[65] The majority of university professors, physicians and government officials, however, generally have internet access, thus minimizing the coverage error.[71, 84] Furthermore, in any survey, including a web-based survey, respondents differ from the non-respondents in terms of demographics and attitude resulting in non-response error.[85] Nonetheless, research reports comparing Internet – such as the one we report in the present survey – and mail survey methodology suggest that differences between responders and non-responders are likely small.[86] In addition, this literature contains no account of response bias based on demographic characteristics. Finally, web-based surveys are susceptible to multiple survey completions by the same person (i.e., "ballot stuffing"). We have implemented certain technological measures, such as the use of cookies and IP addresses, to avoid duplicate responses.[65]

The present survey attempts to estimate the prevalence of attitudes and behaviours in a population of physicians using a self-selected sample. Although it is theoretically possible that the physicians who chose to complete our survey were already those most likely to use placebos, in light of the abovementioned explanations this possibility is unlikely. It is likely however, that our results represent specific trends and capture clinical undercurrents that may be of general interest.

Conclusions

With Internet access becoming ubiquitous, online surveys loom as potentially powerful tools to probe such populations as academic physicians. Web-based technology – such as the one we

used in the present study – is hardly a remedy to all survey research problems. Similar to other tools in a researcher's toolkit, however, Internet surveys fit some tasks better than others. Although such methods require further refinement, using this tool appropriately paves the road to a more scientific way of practicing eHealth.[65]

Although no difference existed between the 20% of physicians reporting that they had either prescribed or administered a placebo, psychiatrists were less likely to report that placebos had no clinical benefit. Overall, psychiatrists' attitudes appear less stringent towards applying placebos in clinical practice.

Defining placebos continues to be a source of debate and confusion within the medical community.[1, 3, 4, 18, 87] This tenuous gray zone probably plays at least some role in obtaining results from over 35% of psychiatrists, who report prescribing subtherapeutic doses without expected clinical efficacy, while considerably fewer of them report prescribing placebos. Although most scholars, by definition, would construe "subtherapeutic doses" as placebo-like, our findings suggest that respondents entertain an inconsistent conceptualization of placebos and their effects. For example, at least some psychiatrists construe subtherapeutic doses as having therapeutic benefits – a scantily addressed issue, which merits further exploration. How can we determine whether a psychiatrist is operating under a therapeutic misconception regarding dose? This is an empirical question for experimental science to answer. While most physicians likely appreciate the clinical merits of placebos, limited guidelines and scientific knowledge, not to mention inadequate ethical considerations, impede open discussion concerning the optimal incorporation of placebos into the medical milieu.

DRAFT

Acknowledgments

We thank the Educational Technologies team at McGill University for their technical support and maintenance of the online survey.

Funding and support

Dr. Raz acknowledges the kind support of the Canada Research Chair program as well as grant support from the Canadian Institute of Health Research, the Natural Sciences and Engineering Research Council of Canada, and the Oxford-McGill Neuroscience Collaboration.

DRAFT

Table 1: Typologies of placebo definitions. Differences regarding options b, c, and d were due to response variation between male psychiatrists and male non-psychiatrists. ♀ = females, ♂ = males.

Item #8: The following statement(s) best describe(s) my definition of a placebo			
**Definition**	**Psychiatrists (%)**	**Other Physicians (%)**	**Result**
a. An intervention that is not expected to have an effect through a known physiological mechanism	46.3<br>♀ 48.9<br>♂ 45.0	55.3<br>♀ 58.9<br>♂ 53.3	$\chi^2(1) = 4.80$, $P = 0.03$<br>♀ $\chi^2(1) = 2.08$, $P = 0.15$<br>♂ $\chi^2(1) = 2.70$, $P = 0.10$
b. An intervention not considered to have any "specific" effect on the condition treated, but with a possible "unspecific" effect	52.9<br>♀ 47.7<br>♂ 55.6	37.3<br>♀ 39.5<br>♂ 36.0	$\chi^2(1) = 14.76$, $P < 0.001$<br>♀ $\chi^2(1) = 1.42$, $P = 0.23$<br>♂ $\chi^2(1) = 15.05$, $P < 0.001$
c. An intervention that is inert or innocuous	19.5<br>♀ 22.7<br>♂ 17.8	32.1<br>♀ 29.0<br>♂ 33.8	$\chi^2(1) = 12.07$, $P < 0.001$<br>♀ $\chi^2(1) = 1.03$, $P = 0.30$<br>♂ $\chi^2(1) = 12.60$, $P < 0.001$
d. Other (alternative definition)	6.7<br>♀ 3.4<br>♂ 8.3	2.6<br>♀ 2.4<br>♂ 2.7	$\chi^2(1) = 5.87$, $P = 0.02$<br>♀ $\chi^2(1) = 0.18$, $P = 0.67$<br>♂ $\chi^2(1) = 6.32$, $P = 0.01$

Percentages may not add up to 100% because each physician could select multiple options.

DRAFT

Table 2

Answers to Item # 11.

**If I were to prescribe a placebo, I would tell the patient that:**	**Psychiatrists (%)**	**Other Physicians (%)**	***P* - value**
It is a medication	5.1	4.9	$\chi^2(1) = 0.01$, $P = 0.92$
It is a placebo	17.5	10.3	$\chi^2(1) = 6.62$, $P = 0.01$
It is medicine with no specific effect	7.0	4.6	$\chi^2(1) = 1.64$, $P = 0.20$
It is a substance that may help and will not harm	31.1	35.5	$\chi^2(1) = 1.28$, $P = 0.26$
I say nothing	2.3	1.4	Fisher exact test, $P = 0.54$
I would never give a placebo (outside of a clinical research trial)	47.1	47.6	$\chi^2(1) = 0.01$, $P = 0.91$
Other	8.2	4.0	$\chi^2(1) = 4.71$, $P = 0.03$

DRAFT

Figure 1. Responses to Item #9: “I have prescribed or given the following form(s) of treatment in situations without demonstrated or expected clinical efficacy”. Although approximately 48% of both psychiatrists and non-psychiatrists reported that they “would never give a placebo outside of a clinical research trial,” a comparable percentage of physicians from both groups prescribed at least one (76%), two (25%), or three (11%) different unwarranted treatments. (Data from non-psychiatrists are in dotted columns.)

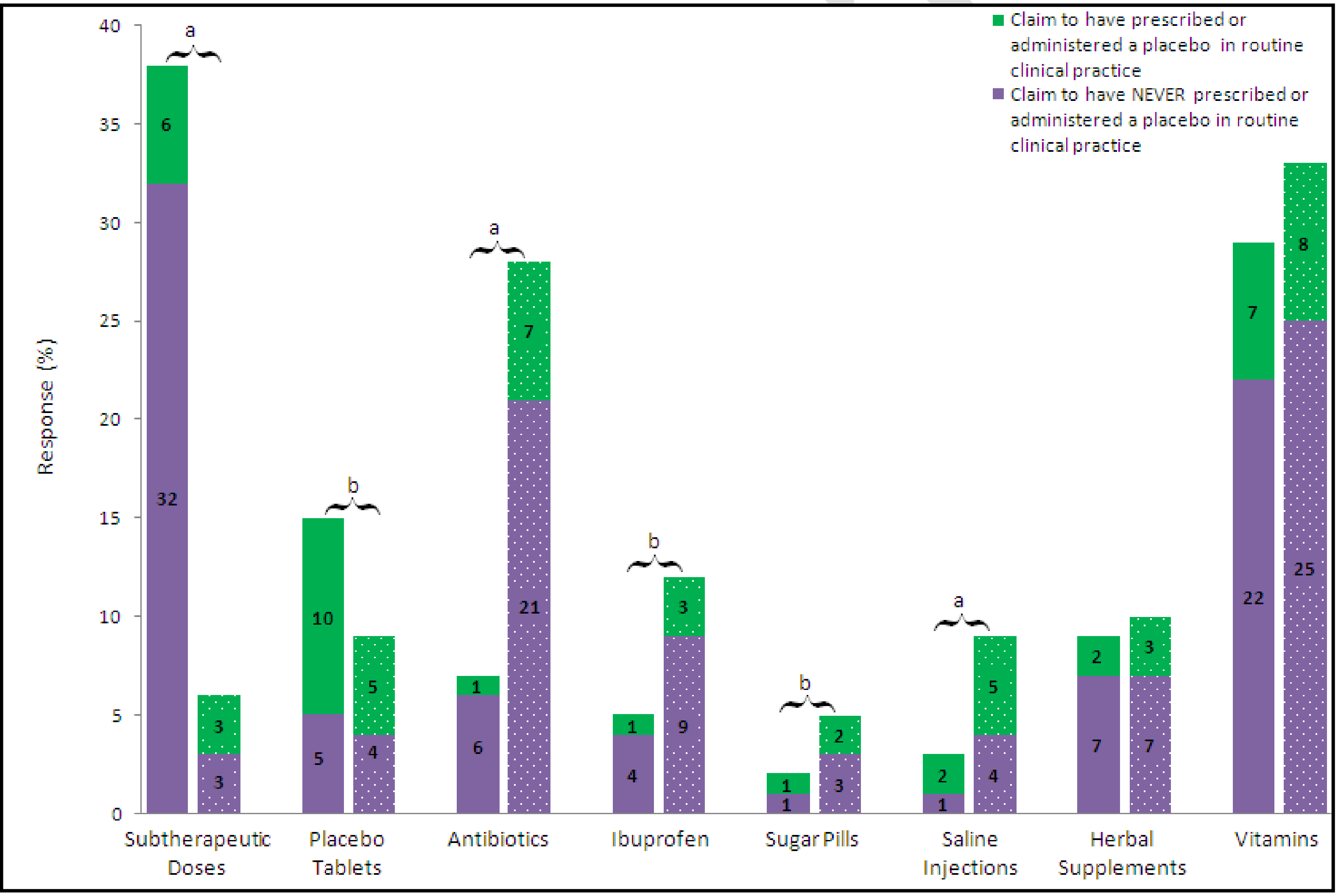

a = $P < 0.001$

b = $P < 0.05$

Figure 2

Percent rating of agreement from psychiatrists (inner circle) and other physicians (outer circle) to Item #13: "I believe placebos have therapeutic effects." Answers ranged from 1 (strongly disagree) to 5 (strongly agree). $\chi^2(4) = 22.74$, $P < 0.001$ between the two groups.

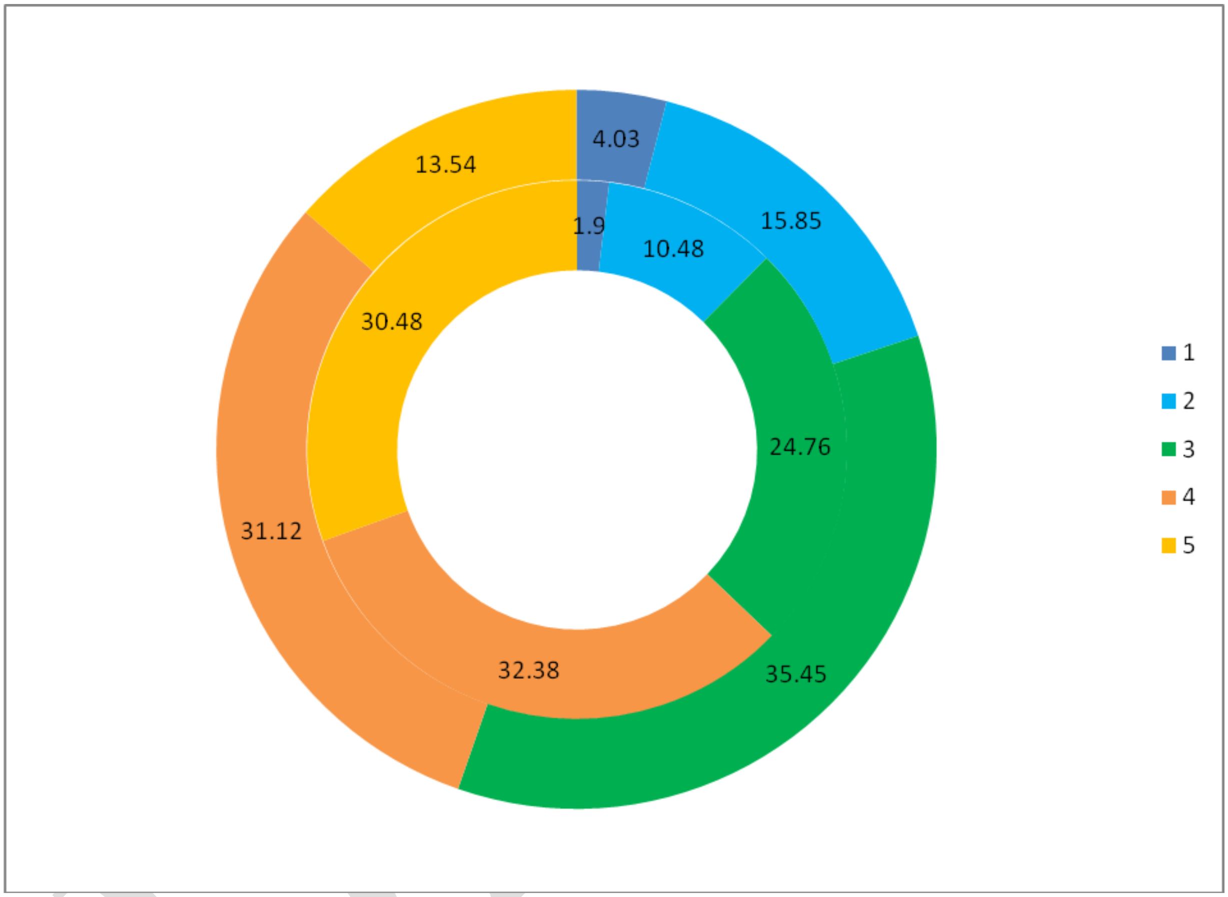

Table 3. Answers to Item #20 and Item # 21. PSYC = Psychological effects; PHYS = Physiological effects; Both = Both PSYC & PHYS; Neither = Neither PSYC nor PHYS.

**20. What benefits can placebo treatments have for the following health problems?**									
**Health Problem**	**Psychiatrists (%)**				**Psychiatrists vs. Non-psychiatrists**	**Other Physicians (%)**			
	**PSYC Only**	**PHYS Only**	**Both**	**Neither**		**PSYC Only**	**PHYS Only**	**Both**	**Neither**
Mental Disorders	18.9	0.4	75.4	5.3	Fisher exact test, $P < 0.001$	36.0	0.7	53.9	9.5
Neurological Disorders	22.3	3.4	62.6	11.7	$\chi^2(3) = 28.23$, $P < 0.001$	30.3	2.1	40.9	26.8
Cancer	28.4	1.9	54.8	14.9	Fisher exact test, $P < 0.001$	39.3	0.7	32.1	27.9
Recovery from Addiction	20.0	1.0	67.6	11.4	Fisher exact test, $P < 0.001$	28.9	1.1	52.8	17.3
Pain	13.9	1.8	82.1	2.2	Fisher exact test, $P < 0.001$	24.6	1.3	63.4	10.7
Immune Problems/ Allergies	17.3	4.1	65.0	13.7	$\chi^2(3) = 46.06$, $P < 0.001$	23.1	2.1	37.0	37.7
Viral Infections	26.5	2.6	43.9	27.0	$\chi^2(3) = 22.20$, $P < 0.001$	31.2	1.4	24.8	42.6
Gastrointestinal Disorders	18.4	1.9	72.0	7.7	Fisher exact test, $P < 0.001$	30.3	1.1	48.9	19.7
Cardiovascular Disorders	22.9	1.6	62.0	13.5	Fisher exact test, $P < 0.001$	27.5	0.7	35.7	36.1
Sleep Disorders	17.5	1.4	76.8	4.3	Fisher exact test, $P < 0.001$	29.6	0.7	58.5	11.2
Sexual Dysfunction	19.7	1.9	70.2	8.2	Fisher exact test, $P < 0.001$	30.9	0.7	53.3	15.1
**21. What types of benefits do you think these categories can have?**									
**Category**									
Meditation, Yoga or Relaxation Techniques	14.4	0.4	82.1	3.1	Fisher exact test, $P = 0.002$	9.1	0.6	89.7	0.6
Hypnosis	16.7	0.0	83.3	0.0	Fisher exact test, $P < 0.001$	23.7	1.3	65.3	9.7
Social Support System	8.0	0.0	92.0	0.0	Fisher exact test, $P = 0.004$	26.4	0.6	72.2	0.9
Good Emotional Health	35.3	0.9	55.1	8.7	Fisher exact test, $P = 0.05$	13.6	0.3	85.5	0.6
Interior Design of Healthcare Environment	20.8	0.4	76.2	2.6	Fisher exact test, $P < 0.001$	43.9	1.3	37.2	17.6
Prayer or Spirituality	21.9	0.0	77.7	0.4	Fisher exact test, $P = 0.008$	31.6	0.6	63.2	4.6
Expectation or Belief	17.8	0.4	81.3	0.4	Fisher exact test, $P < 0.001$	33.4	1.2	62.6	2.7
Doctor-patient Rapport	14.0	0.9	79.3	5.4	Fisher exact test, $P < 0.001$	32.7	1.2	64.9	1.2
Complementary and Alternative Medicine	6.8	4.6	86.8	1.8	Fisher exact test, $P = 0.01$	23.8	1.3	67.0	7.9
Biofeedback	2.5	0.8	96.7	0.0	$\chi^2(3) = 15.98$, $P = 0.001$	14.5	3.2	75.6	6.8

DRAFT

References

1. Harrington A. The many meanings of the placebo effect: Where they came from, why they matter. Biosocieties. 2006;1:181-93.
2. Raz A, Raikhel E, Anbar R. Placebos in Medicine: Knowledge, Beliefs, and Patterns of Use. McGill Journal of Medicine: MJM. 2008;11(2):206.
3. Harrington A. The placebo effect : an interdisciplinary exploration. Cambridge, Mass.: Harvard University Press; 1997.
4. Shapiro AK, Shapiro E. The powerful placebo : from ancient priest to modern physician. Baltimore: Johns Hopkins University Press; 1997.
5. Wager TD, Scott DJ, Zubieta J-K. Placebo effects on human {micro}-opioid activity during pain. Proceedings of the National Academy of Sciences. 2007 June 26, 2007;104(26):11056-61.
6. Wager TD, Rilling JK, Smith EE, Sokolik A, Casey KL, Davidson RJ, et al. Placebo-Induced Changes in fMRI in the Anticipation and Experience of Pain. Science (New York, N.Y. 2004 February 20, 2004;303(5661):1162-7.
7. Raz A, Buhle J. Typologies of attentional networks. Nat Rev Neurosci. 2006;7(5):367-79.
8. Benedetti F. Placebo effects : understanding the mechanisms in health and disease. Oxford ; New York: Oxford University Press; 2008.
9. Frank JD, Frank J. Persuasion and healing : a comparative study of psychotherapy. 3rd ed. Baltimore: Johns Hopkins University Press; 1991.
10. Brown WA. Understanding and Using the Placebo Effect. Psychiatric Times. 2006;23(11):15-7.
11. Fournier JC, DeRubeis RJ, Hollon SD, Dimidjian S, Amsterdam JD, Shelton RC, et al. Antidepressant Drug Effects and Depression Severity: A Patient-Level Meta-analysis. JAMA. 2010;303(1):47-53.
12. Kirsch I, Deacon BJ, Huedo-Medina TB, Scoboria A, Moore TJ, Johnson BT. Initial severity and antidepressant benefits: a meta-analysis of data submitted to the Food and Drug Administration. PLoS Med. 2008 Feb;5(2):e45.
13. Kirsch I, Moore TJ, Scoboria A, Nicholls SS. The Emperor's New Drugs: An Analysis of Antidepressant Medication Data Submitted to the U.S. Food and Drug Administration. Prevention & Treatment. 2002;5(1).
14. Khan A, Kolts RL, Rapaport MH, Krishnan KRR, Brodhead AE, Browns WA. Magnitude of placebo response and drug-placebo differences across psychiatric disorders. Psychological Medicine. 2005;35(5).
15. Kirsch I, Sapirstein G. Listening to Prozac but Hearing Placebo: A Meta-Analysis of Antidepressant Medication. Prevention & Treatment. 1998;1(2).
16. Antonuccio DO, Danton WG, DeNelsky GY, Greenberg RP, Gordon JS. Raising Questions about Antidepressants. Psychother Psychosom 1999;68(1):3–14.
17. Antonuccio DO, Burns DD, Danton WG. Antidepressants: A Triumph of Marketing Over Science? Prevention & Treatment. 2002;5(1).
18. Raz A, Guindi D. Placebos and Medical Education. McGill Journal of Medicine. 2008;11(2): 223–6.
19. CEJA. Placebo use in Clinical practice. 2006.

DRAFT

20. Hrobjartsson A, Gotzsche PC. Is the placebo powerless? An analysis of clinical trials comparing placebo with no treatment. N Engl J Med. 2001 May 24;344(21):1594-602.
21. Greene PJ, Wayne PM, Kerr CE, Weiger WA, Jacobson E, Goldman P, et al. The powerful placebo: doubting the doubters. Adv Mind Body Med. 2001;17(4):298-307; discussion 12-8.
22. Kirsch I, Scoboria A. Apples, oranges, and placebos: heterogeneity in a meta-analysis of placebo effects. Adv Mind Body Med. 2001;17(4):307-9.
23. Spiegel D, Kraemer H, Carlson RW. Is the placebo powerless. N Engl J Med. 2001;345:1276.
24. Price DD, Finniss DG, Benedetti F. A comprehensive review of the placebo effect: recent advances and current thought. Annu Rev Psychol. 2008;59:565-90.
25. Wampold BE, Minami T, Tierney SC, Baskin TW, Bhati KS. The placebo is powerful: estimating placebo effects in medicine and psychotherapy from randomized clinical trials. J Clin Psychol. 2005;61(7):835-54.
26. Hrobjartsson A, Gotzsche PC. Powerful spin in the conclusion of Wampold et al.'s re-analysis of placebo versus no-treatment trials despite similar results as in original review. J Clin Psychol. 2007;63(4):373-7.
27. Wampold BE, Imel ZE, Minami T. The placebo effect: "Relatively large" and "robust" enough to survive another assault. J Clin Psychol. 2007;63(4):401-3.
28. Hrobjartsson A, Gotzsche PC. Is the placebo powerless? Update of a systematic review with 52 new randomized trials comparing placebo with no treatment. Journal of Internal Medicine. 2004;256:91-100.
29. Kolber AJ. A Limited Defense of Clinical Placebo Deception. Yale Law & Policy Review. 2007;26(1):75-134.
30. Foddy B. A duty to deceive: placebos in clinical practice. American Journal of Bioethics. 2009;in review.
31. Miller F, Colloca L. The legitimacy of placebo treatments in clinical practice: evidence and ethics. The American journal of bioethics: AJOB. 2009;9(12):39.
32. Lichtenberg P. The Placebo: Pervasive and Permissible. McGill Journal of Medicine. In press.
33. Benedetti F. Mechanisms of Placebo and Placebo-Related Effects Across Diseases and Treatments. Annual review of pharmacology and toxicology. 2008;48(1):33-60.
34. Price DD, Finniss DG, Benedetti F. A comprehensive review of the placebo effect: recent advances and current thought. Annu. Rev. Psychol. 2008;59.
35. Harrington A. The cure within : a history of mind-body medicine. 1st ed. New York: W.W. Norton; 2008.
36. Harris G. Half of Doctors Routinely Prescribe Placebos. The New York Times. 2008 October 23.
37. Rubin R. Placebo effect: New survey gives life to ethical debate. USA Today. 2008.
38. Moerman DE. Meaning, medicine, and the "placebo effect". Cambridge, U.K. ; New York: Cambridge University Press; 2002.
39. Moerman DE, Jonas WB. Deconstructing the placebo effect and finding the meaning response. Annals of internal medicine. 2002 Mar 19;136(6):471-6.
40. Moerman D. Doctors and patients: the role of clinicians in the placebo effect. Advances in Mind-Body Medicine. 2003 Spring;19(1):14-22.

DRAFT

41. Thompson JJ, Ritenbaugh C, Nichter M. Reconsidering the placebo response from a broad anthropological perspective. Culture, Medicine and Psychiatry. 2009;33(1):112-52.
42. Tilburt JC, Emanuel EJ, Kaptchuk TJ, Curlin FA, Miller FG. Prescribing "placebo treatments": results of national survey of US internists and rheumatologists. BMJ. 2008;337:a1938.
43. Goldberg RJ, Leigh H, Quinlan D. The current status of placebo in hospital practice. General Hospital Psychiatry. 1979;1(3):196-201.
44. Goodwin JS, Goodwin JM, Vogel AV. Knowledge and Use of Placebos by House Officers and Nurses. Annals of internal medicine. 1979 07;91(1):106.
45. Gray G, Flynn P. A survey of placebo use in a general hospital. General Hospital Psychiatry. 1981;3(3):199-203.
46. Ernst E, Abbot NC. Placebos in clinical practice: results of a survey of nurses. Perfusion. 1997;10:128-30.
47. Hrobjartsson A, Norup M. The Use of Placebo Interventions in Medical Practice--A National Questionnaire Survey of Danish Clinicians. Eval Health Prof. 2003 June 1, 2003;26(2):153-65.
48. Nitzan U, Lichtenberg P. Questionnaire survey on use of placebo. BMJ. 2004 October 23, 2004;329(7472):944-6.
49. Sherman R, Hickner J. Academic physicians use placebos in clinical practice and believe in the mind-body connection. J Gen Intern Med. 2008 Jan;23(1):7-10.
50. Laporte JR, Figueras A. Placebo effects in psychiatry. Lancet. 1994;344(8931):1206-9.
51. Ernst E. Towards a scientific understanding of placebo effects. In: Peters D, editor. Understanding the placebo effect in complementary medicine: theory, practice and research. London: Churchill Livingstone; 2001.
52. Miller FG, Emanuel EJ, Rosenstein DL, Straus SE. Ethical Issues Concerning Research in Complementary and Alternative Medicine. JAMA. 2004 February 4, 2004;291(5):599-604.
53. Ernst E. Placebo: new insights into an old enigma. Drug Discovery Today. 2007;12(9-10):413-8.
54. Ernst E. Placebo forte. Wiener Medizinische Wochenschrift. 1992;142:217-9.
55. Campbell N, editor. Patterns and misconceptions in the use of clinical placebos. 1st Department of Psychiatry Student Research Day; June 10th 2009; Jewish General Hospital (ICFP), Montreal, Quebec.
56. Lichtenberg P, Heresco-Levy U, Nitzan U. The ethics of the placebo in clinical practice. J Med Ethics. 2004;30(6):551-4.
57. Kirsch I, Deacon BJ, Huedo-Medina TB, Scoboria A, Moore TJ, Johnson BT. Initial severity and antidepressant benefits: A meta-analysis of data submitted to the food and drug administration. PLoS medicine. 2008;5(2):0260-8.
58. Kirsch I. The Emperor's New Drugs: Exploding the Antidepressant Myth. London: Bodley Head; 2009.
59. Fournier JC, DeRubeis RJ. For whom do antidepressant medications work? The effects of antidepressants across the range of symptom severity. Psychologie Québec: Integrating Science and Practice. in press.
60. Fulton MM, Allen ER. Polypharmacy in the elderly: a literature review. JAANP. 2005;17(4):123-32.
61. Silberman S. Placebos Are Getting More Effective. Drugmakers Are Desperate to Know Why. Wired Magazine, 08.24.09. 2009.

DRAFT

62. Beaumont G, Baldwin D, Lader M. A criticism of the practice of prescribing subtherapeutic doses of antidepressants for the treatment of depression. Human Psychopharmacology. 1996;11(4):283-91.
63. Fairman KA, Drevets WC, Kreisman JJ, Teitelbaum F. Course of antidepressant treatment, drug type, and prescriber's specialty. Psychiatric Services. 1998;49(9):1180-6.
64. Hartung DM, Wisdom JP, Pollack DA, Hamer AM, Haxby DG, Middleton L, et al. Patterns of atypical antipsychotic subtherapeutic dosing among Oregon Medicaid patients. Journal of Clinical Psychiatry. 2008;69(10):1540-7.
65. Couper MP. Issues of Representation in eHealth Research (with a Focus on Web Surveys). Am J Prev Med. 2007;32(5):S83-S9.
66. Couper MP, Miller PV. Web Survey Methods: Introduction. Public Opin Q. 2008 December 1, 2008;72(5):831-5.
67. Callegaro M, DiSogra C. Computing Response Metrics for Online Panels. Public Opin Q. 2008 December 1, 2008;72(5):1008-32.
68. Standard Definitions: Final Dispositions of Case Codes and Outcome Rates for Surveys. The American Association for Public Opinion Research 2008 [cited 2009 May 20]; Available from: http://www.aapor.org/responseratesanoverview.
69. Bosnjak M, Neubarth W, Couper MP, Bandilla W, Kaczmirek L. Prenotification in Web-Based Access Panel Surveys: The Influence of Mobile Text Messaging Versus E-Mail on Response Rates and Sample Composition. Soc Sci Comput Rev. 2007 December 3, 2007:0894439307305895.
70. Underwood D, Kim, H., Matier, M. To Mail or To Web: Comparisons of Survey Response Rates and Respondent Characteristics. Paper presented at the Annual Forum of the Association for Institutional Research; Cincinnati, OH: (ERIC Reproduction Service No. ED446513); 2000.
71. Dillman DA. Mail and Internet surveys : The Tailored design method 2nd edition ed. New York: John Wiley and Sons; 2000.
72. Manfreda KL, Bosnjak M, Berzelak J, Haas I, Vehovar V. Web surveys versus other survey modes: A meta-analysis comparing response rates. Int J Market Res. 2008;50(1):79-104.
73. Matz CM. Administration of Web versus Paper Surveys: Mode Effects and Response Rates. Masters research paper. Chapel Hill: University of North Carolina; 1999.
74. Crawford SD, Couper MP, Lamias MJ. Web surveys: Perceptions of burden. Soc Sci Comput Rev. 2001;19(2):146-62.
75. Smith CB. Casting the net: Surveying an Internet population. JCMC. 1997;3(1).
76. Tse ACB, Tse KC, Yin CH, Ting CB, Yi KW, Yee KP, et al. Comparing two methods of sending out questionnaires: E-mail versus mail. J Market Res Soc. 1995;37(4):441-6.
77. Witmer DF, Coleman, R.W. & Katzman, S.L. From paper-and-pencil to screen-and-keyboard: Toward a methodology for survey research on the Internet. In: Jones S, editor. Doing Internet research: Critical issues and methods for examining the Net. Thousand Oaks, Calif. : Sage Publications; 1999.
78. Cook C, Heath F, Thompson RL. A meta-analysis of response rates in Web- or internet-based surveys. Educational and Psychological Measurement. 2000;60(6):821-36.
79. Krosnick J. Survey research. Annual Review of Psychology. 1999;50(1):537-67.
80. Canadian Institute for Health Information. *Supply, distribution and migration of Canadian physicians, 2007*. (Ottawa, Ont: CIHI, 2008).

DRAFT

81. Eysenbach G, Wyatt J. Using the Internet for Surveys and Health Research. J Med Internet Res 2002;4(2):e-13.
82. Pealer LN, Weiler RM, Pigg RM, Jr., Miller D, Dorman SM. The Feasibility of a Web-Based Surveillance System to Collect Health Risk Behavior Data from College Students. Health Educ Behav. 2001 October 1, 2001;28(5):547-59.
83. Couper MP, Tourangeau R, Steiger DM. Social presence in Web surveys. Proceedings of the SIGCHI conference on Human factors in computing systems; Seattle, Washington, United States: ACM; 2001.
84. Martin S. MDs' office Internet use hits 57%. CMAJ. 2003 February 18, 2003;168(4):475-a-.
85. Umbach PD. Web surveys: Best practices. New Directions for Institutional Research. 2004;2004(121):23-38.
86. Sax LJ, Gilmartin, S. K., Hagedorn, L. S., Lee, J. J. Using Web Surveys to Reach Community College Students: An Analysis of Response Rates and Response Bias. Community College Journal of Research and Practice. 2008;32(9):712-29.
87. Kaptchuk TJ. The double-blind, randomized, placebo-controlled trial: Gold standard or golden calf? J Clin Epidemiol. 2001;54(6):541-9.

DRAFT
